# Ancient permafrost staphylococci carry antibiotic resistance genes

**DOI:** 10.1080/16512235.2017.1345574

**Published:** 2017-01-01

**Authors:** Elena Kashuba, Alexey A. Dmitriev, Shady Mansour Kamal, Ojar Melefors, Gennady Griva, Ute Römling, Ingemar Ernberg, Vladimir Kashuba, Anatoli Brouchkov

**Affiliations:** ^a^ Department of Microbiology, Tumor and Cell Biology (MTC), Karolinska Institutet, Stockholm, Sweden; ^b^ Laboratory of Cancer Genetics, R.E. Kavetsky Institute of Experimental Pathology, Oncology and Radiobiology, NASU, Kyiv, Ukraine; ^c^ Engelhardt Institute of Molecular Biology, Russian Academy of Sciences, Moscow, Russia; ^d^ Department of Microbiology and Immunology, Faculty of Pharmaceutical Sciences & Pharmaceutical Industries, Future University in Egypt, New Cairo, Egypt; ^e^ Siberian Branch of RAS, Institute of the Earth Cryosphere, Tyumen, Russia; ^f^ Department of Molecular Oncogenetics, Institute of Molecular Biology and Genetics, NASU, Kyiv, Ukraine; ^g^ Faculty of Geology, Lomonosov Moscow State University, Moscow, Russia

**Keywords:** Permafrost, genome sequence, evolution, antibiotic resistance, microbial physiology, biofilm, *Staphylococcus*

## Abstract

**Background**: Permafrost preserves a variety of viable ancient microorganisms. Some of them can be cultivated after being kept at subzero temperatures for thousands or even millions of years.

**Objective**: To cultivate bacterial strains from permafrost.

**Design**: We isolated and cultivated two bacterial strains from permafrost that was obtained at Mammoth Mountain in Siberia and attributed to the Middle Miocene. Bacterial genomic DNA was sequenced with 40–60× coverage and high-quality contigs were assembled. The first strain was assigned to *Staphylococcus warneri* species (designated MMP1) and the second one to *Staphylococcus hominis* species (designated MMP2), based on the classification of 16S ribosomal RNA genes and genomic sequences.

**Results**: Genomic sequence analysis revealed the close relation of the isolated ancient bacteria to the modern bacteria of this species. Moreover, several genes associated with resistance to different groups of antibiotics were found in the *S. hominis* MMP2 genome.

**Conclusions**: These findings supports a hypothesis that antibiotic resistance has an ancient origin. The enrichment of cultivated bacterial communities with ancient permafrost strains is essential for the analysis of bacterial evolution and antibiotic resistance.

## Introduction

Psychrotrophs and psychrophiles are microorganisms that can survive or even thrive in cold places [1,2]. Investigations of glacier ice at the Vostok station in the Antarctic [3] and surface snow from the South Pole [4] revealed that bacteria, fungi, diatoms, and other microorganisms were probably trapped in the snow and remained in the ice for thousands of years. Microbial activity was also found in ice sediments of perennial and permanent lake ice [5,6] and in the Antarctic Sea [7,8]. Anaerobic microorganisms were isolated from glacier samples in the Arctic [9,10] and Greenland [11].

Permafrost (permafrost soil) is soil that stays at 5–10ºC below zero for 2 years or longer. Most of the permafrost is located in lands close to the North and South Poles. However, some permafrost exists at much lower latitudes. A thin active layer of the soil above the permafrost usually thaws during the summer. The thickness of this active layer varies depending on season and location, but is typically 0.6–4.0 m. In the northern Lena and Yana River basins in Siberia (Russia), the permafrost depth may be more than 1400 m. The structure of permafrost is very stable, with nothing changing for thousands and even millions of years. The permafrost environment is considered extreme because of subzero temperatures and background radiation over geological timescales. The age of permafrost can be proven by its history of freezing, geological conditions, and radioisotope dating [12,13].

Permafrost sediments contain microorganisms [14–17]. Viable bacterial strains have been isolated from 25,000-year-old ice wedges from Alaska [12,18,19]. These bacteria, which were isolated from the rest of the world for a long period (thousands and millions of years), are invaluable for studies on modern diseases and the evolutionary history of microorganisms.

Ancient viable microorganisms were claimed to have been be cultured from Dominican amber, considered to be 20–40 million years old [20]. In our previous collaborative work, viable bacteria were obtained from permafrost of the Mammoth Mountain, Siberia, aged 40,000 to 4 million years depending on the sampling depth [13,16,21]. Moreover, the genomic DNA of one strain of bacteria, identified as *Bacillus cereus*, was isolated and sequenced [22].

In the present work, we aimed to enrich the number of viable bacteria from permafrost, study their morphology, and analyze the genomic sequences.

## Material and methods

### Permafrost samples and microorganism cultivation

In this article, we describe bacterial strains isolated from permafrost in the River Lena region in Central Yakutia, Russia. According to the composition of seeds, pollen, and leaves, this permafrost is of Middle Miocene, and thus the permafrost may be up to 3.5 million years old [21]. Permafrost samples were taken from an exposure, due to river erosion, on the Mammoth Mountain on the Aldan River, as described in detail previously [21].

In brief, permafrost samples were taken in July 2009 at a depth of about 50 m below the surface and below the active layer of 1 m. Samples of the frozen soil were cut with a handsaw or a circular saw and preserved at a temperature of about −15°C. Permafrost was kept frozen until delivery to the laboratory at the Department of Microbiology, Tumor and Cell Biology, Karolinska Institutet (MTC). Collected material (approximately 10 cm^3^) was kept at −20°C at MTC. Smaller blocks of permafrost were cut out using a sterile handsaw at −20°C (in the freeze room at MTC). These blocks were fragmented under sterile conditions using a sterile hammer. Small portions of still-frozen soil were drilled out of the bigger block in a laminar flow cabinet, after sterilization of the cabinet by an ultraviolet-C germicidal lamp for 15 min. These samples were bathed in 90% ethanol and put in Lauri–Bertani (LB) medium (about 6 g of soil in 5 ml of LB medium). A suspension possibly containing microorganisms was plated on a large (14.5 cm diameter) Petri dish with rich yeast extract peptone dextrose (YPD) medium without glucose. Two LB plates with no suspension were used as a negative control. Plates were incubated at 37°C. Two different colonies were isolated on 18 January 2010. Both strains were cultured individually, on agar plates and in liquid LB medium supplemented with glucose (2% w/v). No colonies were observed on the control plates.

### Transmission electron microscopy

Cells were grown in tryptic soy broth (TSB) at 37°C overnight. Fixation was achieved in a mixture of 2% glutaraldehyde and 1% formaldehyde in 0.1 M phosphate-buffered saline (PBS), pH 7.4, at room temperature and stored at +4ºC. After fixation, cells were rinsed in 0.1 M PBS and centrifuged. The pellets were then post-fixed in 2% osmium tetroxide (TAAB, Aldermaston, UK) solution in 0.1 M PBS at 4°C for 2 h, dehydrated in ethanol followed by acetone, and embedded in LX-112 (Ladd, Williston, VT, USA). The ultrathin sections (approximately 50–60 nm) were cut by a Leica Ultracut UCT (Leica, Vienna, Austria). Sections were contrasted with uranyl acetate followed by lead citrate and examined in a Hitachi HT 7700 (Hitachi, Tokyo, Japan) at 80 kV. Digital images were taken with a Veleta camera (Olympus Soft Imaging Solutions, Münster, Germany).

### Biofilm formation

The ability of bacteria to form biofilms was assessed on Congo red (CR) agar, as described previously [23]. In brief, bacterial strains were grown in TSB medium overnight. For all strains, the cell density was adjusted to optical density (OD) = 1 and 10 μl of cell suspension was plated on a CR agar plate (3% TSB, 0.75% glucose, 0.008% CR dye, and 1% agar). Cells were grown at 37°C for 17 h. The black color of the grown colony indicates biofilm formation, while a reddish or pinkish color indicates no or less biofilm. For the quantitative studies on biofilms, experiments were performed as described previously [24]. In brief, strains were grown overnight in TSB and diluted to an OD of 0.01 in TSB the next morning. For each strain, 200 μl of the diluted culture was loaded into 16 wells of a flat-bottomed 96-well microtiter plate (TPP, Trasadingen, Switzerland). Following 24 h of incubation at 37°C, the cell density was measured at *λ* = 595 nm. Wells were carefully emptied and washed with the physiological saline. Biofilm attached to the wells was stained with 250 μl of 0.4% crystal violet (CV) for 10 min. After removing CV and washing, the dye was dissolved in 250 μl of 30% acetic acid, and absorbance at *λ* = 595 nm was measured. Relative biofilm formation was calculated as the ratio of the absorbance of the CV-stained biofilm matrix to the cell density at λ = 595 nm.

### Genomic sequencing

Bacterial genomic DNA was isolated from liquid cultures originating from a single colony using a PowerSoil® DNA Isolation Kit (MO BIO Labs, Carlsbad, CA, USA) on 15 February 2010. The quality and concentration of the extracted DNA was verified using a NanoDrop™ spectrophotometer (Thermo Fisher Scientific, Waltham, MA, USA) and by electrophoresis in the 0.8% agarose gel.

Next, 500 ng of DNA from each strain was used for Roche 454 library preparation according to the manufacturer’s protocol (www.454.com) and sequenced in one or two lanes of a four-region FLX Titanium picotiter plate on a Roche 454 FLX instrument (Roche, Basel, Switzerland).

### Sequence assembly

Genome *de novo* assembly was performed using a Roche GS De Novo Assembler (version 2.7) with default parameters. Alignments to reference genomes were performed with a Roche GS Reference Mapper (version 2.7) with default parameters.

### Classification

The 16S ribosomal RNA (rRNA) genes were classified using SINA Alignment Service version 1.2.11 [25] with default parameters. NCBI Microbial Nucleotide BLAST [26] with *de novo* assembled contigs, database of draft genomes, and the Megablast algorithm was also applied.

### Genome annotation

Genome annotation was performed using NCBI Prokaryotic Genome Automatic Annotation Pipeline (PGAAP) [27]. Plasmid sequences were identified with PlasmidFinder version 1.3 [28]. The identity threshold was set to 85%.

### Phylogenetic analysis

We used MEGA version 6.06 [29] and maximum likelihood method with default parameters for phylogenetic tree building on the basis of 16S rRNA sequences. Known 16S rRNA sequences were obtained from the SILVA database [30]. The MUSCLE algorithm was applied for sequence alignment. Bootstrap values were calculated based on 1000 replications.

### Detection of antibiotic resistance and virulence genes

The search for antibiotic resistance and virulence genes was performed with ResFinder version 2.1 [31] and VirulenceFinder version 1.5 [32] web services. The identity threshold was set to 85% and the minimum length to 40% for both tools.

## Results

### Phenotype characteristics

**Figure 1.**
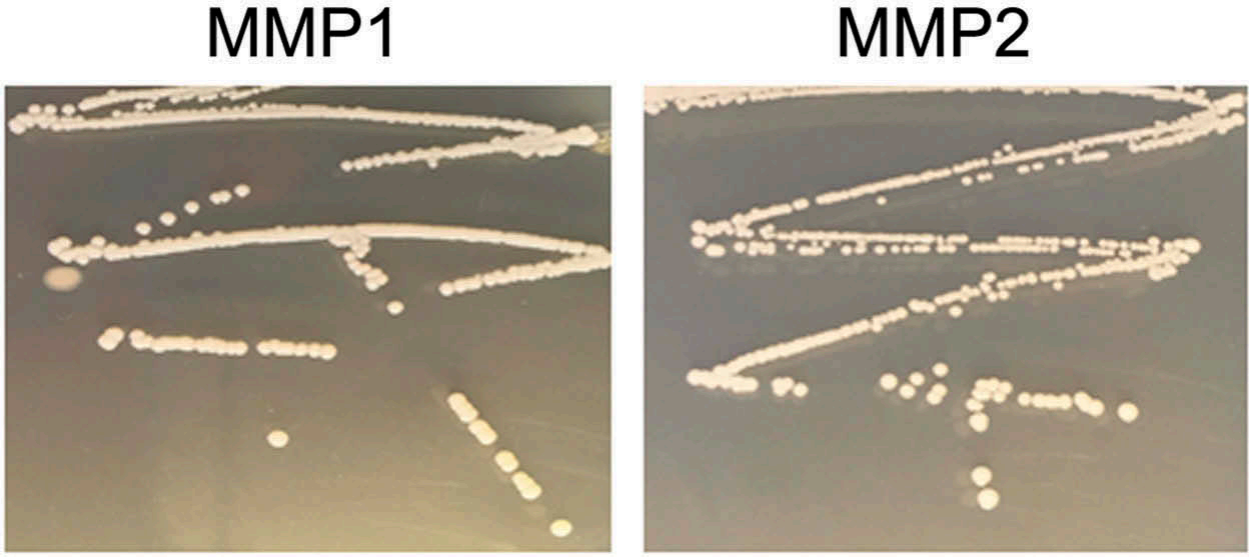
Morphology of colonies on tryptic soy agar plates. Mammoth Mountain Permafrost 1 (MMP1, *Staphylococcus warneri*) and MMP2 (*Staphylococcus hominis*) display as whitish to yellowish smooth round colonies.

The novel strains of microorganisms, which were called MMP1 (Mammoth Mountain Permafrost 1) and MMP2 (Mammoth Mountain Permafrost 2), showed an ability to proliferate under ordinary culturing conditions, both on agar plates and in liquid LB medium. On LB agar plates, both strains produced opaque colonies, which were slightly rough and had wavy edges. MMP1 and MMP2 displayed as whitish to yellowish smooth round colonies on tryptic soy agar (TSA) plates (Figure 1). Cultivation was carried out at 37°C and with intensive aeration (shaking) to reach the logarithmic phase of growth. The cultures were maintained by reseeding on agar plates and were also stored with glycerol (40%) at −80°C.

**Figure 2.**
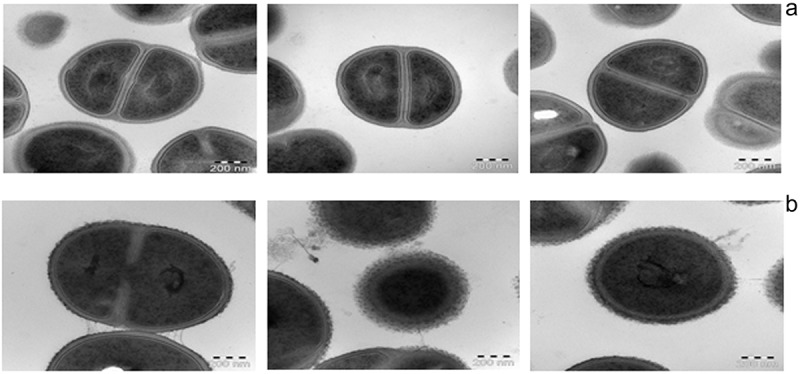
Ultrastructural analysis by thin-section transmission electron microscopy from cells in the stationary phase. (A) Mammoth Mountain Permafrost 1 (MMP1, *Staphylococcus warneri*) cells have a smooth surface and the majority of the cell population shows a fully formed septum (having just divided). (B) MMP2 (*Staphylococcus hominis*) cells have an irregular cell surface and few cells have formed a septum.

MMP1 cells had a smooth surface and the majority of the cell population showed a fully formed septum (Figure 2(a)). MMP2 had an irregular cell surface and few cells formed the septum (Figure 2(b)), as shown by ultrastructural analysis of bacteria grown up to the stationary phase by TEM.

### Biofilm formation

**Figure 3.**
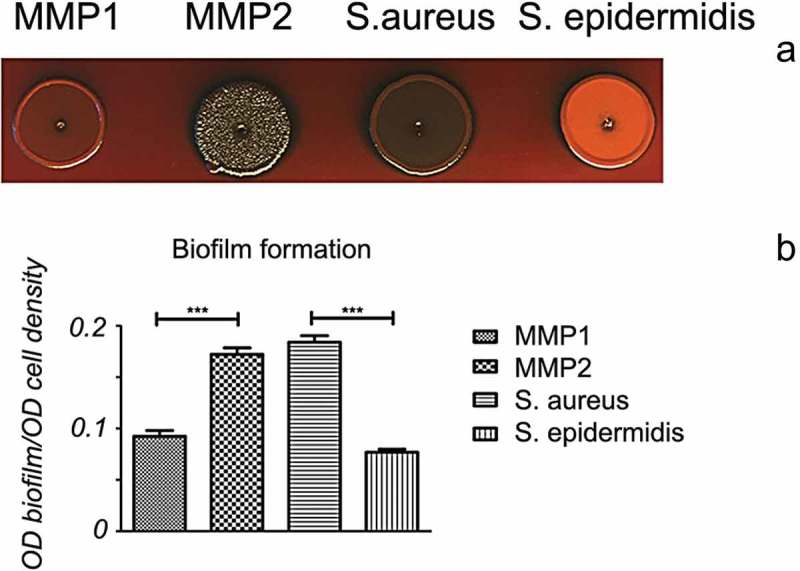
Mammoth Mountain Permafrost 1 (MMP1, *Staphylococcus warneri*) and MMP2 (*Staphylococcus hominis*) can form biofilms. (A) Colony morphology of bacteria on Congo red agar plates. The reddish smooth colony morphology of MMP1 indicates less biofilm formation; the black rough colony of MMP2 indicates high biofilm formation. *Staphylococcus aureus SC08* and *S. epidermidis ATCC 12228* are positive and negative controls, respectively. (B) Quantitative assay on biofilm formation in 96-well plates. Relative biofilm formation shows that MMP1 forms less biofilm than MMP2 (*p* < 0.05). *Staphylococcus aureus SC08* and *S. epidermidis ATCC 12228* are positive and negative controls, respectively. OD, optical density.

To assess the ability of ancient bacteria to form biofilm, MMP1 and MMP2 were grown on CR plates. For MMP1, the formation of the reddish smooth colonies was observed (Figure 3(a)), indicating biofilm formation. MMP2 showed a greater ability to form biofilms, as can be concluded from the appearance of the black rough colonies (Figure 3). Bacterial strains which had previously been characterized for their biofilm formation capacity, i.e. *Staphylococcus aureus SC08* and *S. epidermidis ATCC 12228* [33], served as the positive and negative controls, respectively.

These data were confirmed by a relative biofilm formation assay in 96-well plates (Figure 3(b)). MMP1 formed less biofilm than MMP2, and this difference was statistically significant (*p* < 0.05).

### Sequencing data on the ancient strains

For the ancient strain MMP1, 421.5 k reads with average length of 361 bases were obtained. The reads were assembled in 56 contigs (35 large ones) with total length equal to 2.44 M bases. The sequencing coverage was about 60×. For the ancient strain MMP2, 272.8 k reads with an average length of 323 bases were obtained. The reads were assembled in 84 contigs (65 large ones) with total length equal to 2.21 M bases. Hence, the sequencing coverage was about 40×. The N50 value was 171.3 k bases for strain MMP1 and 92.5 k bases for strain MMP2, indicating that the assemblies were of a high quality. The detailed information is presented in Table 1.

**Table 1.** Summary of *de novo* assembly of the two ancient strains.

Feature	Strain MMP1	Strain MMP2
Total reads	421,504	272,754
Total bases	152,337,898	88,093,644
Average read length	361	323
Aligned reads	416,749 (98.9%)	270,165 (99.1%)
Aligned bases	150,511,972 (98.8%)	87,169,378 (99.0%)
Large contigs	35	65
Bases in large contigs	2,438,922	2,205,320
All contigs	56	84
Bases in all contigs	2,442,906	2,209,742
N50	171,280	92,490
L50	4	8
Sequencing coverage	~ 60×	~ 40×

MMP1, Mammoth Mountain Permafrost 1; MMP2, Mammoth Mountain Permafrost 2.

### Classification

Contigs containing 16S rRNA genes of the ancient strains were found using NCBI BLAST: #27 (1619 bases) for MMP1 and #59 (1541 bases) for MMP2. Based on 16S rRNA gene sequences, both bacteria were classified by SINA as being of genus *Staphylococcus*. The nearest neighbors for strain MMP1 were strains of *S. warneri* and *S. pasteuri* species (*S. warneri* group), and the nearest neighbors for strain MMP2 were strains of *S. haemolyticus* and *S. hominis* species (*S. haemolyticus* group). The same results were obtained using the *de novo* assembled contigs and NCBI Microbial Nucleotide BLAST through *Staphylococcus* group (taxid: 90964). To determine the species of the examined strains more precisely, the raw reads of strain MMP1 were aligned to NCBI representative genomes *S. warneri* SG1 and *S. pasteuri* SP1. The same procedure was performed for strain MMP2 and NCBI representative genomes *S. hominis* subsp. *hominis* C80 and *S. haemolyticus* JCSC1435. All representative genomes are complete genomes, except for *S. hominis* subsp. *hominis* C80, which is a scaffold. The results of the mapping are summarized in Table 2.

**Table 2.** Summary of reference mapping for the two ancient *Staphylococcus* (*S*.) strains.

	*S*. strain MMP1		*S*. strain MMP2	
Feature	*S. warneri* SG1	*S. pasteuri* SP1	*S. hominis* subsp. *hominis* C80	*S. haemolyticus* JCSC1435
Mapped reads	397,917 (94.4%)	377,756 (89.6%)	235,160 (86.2%)	108,373 (39.7%)
Mapped bases	142,222,185 (93.4%)	134,746,471 (88.5%)	75,170,689 (85.3%)	16,664,461 (18.9%)
Large contigs	53	156	107	152
Bases in large contigs	2,315,018	2,252,460	2,018,054	171,102
All contigs	66	212	161	1563
Bases in all contigs	2,318,257	2,266,066	2,030,477	435,147
N50	113,255	27,262	38,136	1,208

MMP1, Mammoth Mountain Permafrost 1; MMP2, Mammoth Mountain Permafrost 2.

**Figure 4.**
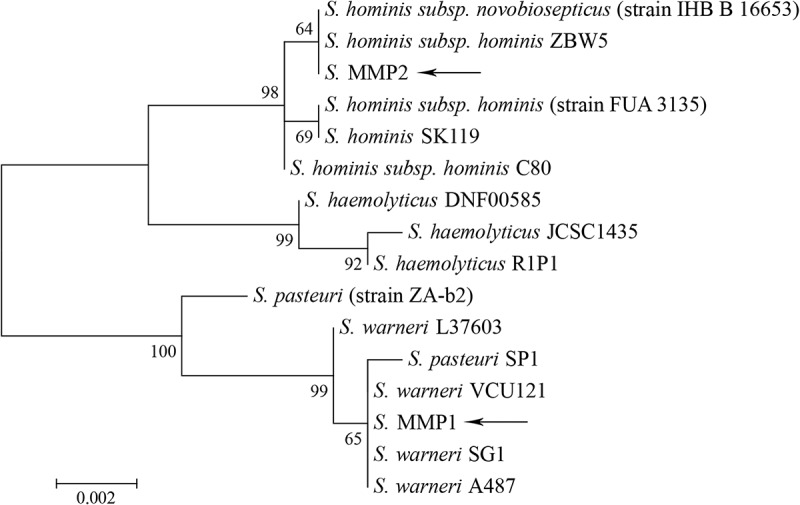
Phylogenetic tree based on 16S ribosomal RNA gene sequences of ancient and modern *Staphylococcus* strains. The ancient strains are marked by arrows. The maximum likelihood method was used for the phylogenetic analysis. Bootstrap values are shown next to the branches. The tree is drawn to scale, with branch lengths measured in the number of substitutions per site. All positions containing gaps and missing data were eliminated. There were 1169 positions in total in the final data set.

As seen from Table 2, strain MMP1 showed a significantly higher similarity to the reference *S. warneri* SG1 compared to *S. pasteuri* SP1 (93.4% vs 88.5% of reads were mapped), and strain MMP2 showed a significantly higher similarity to reference *S. hominis*subsp. *hominis* C80 compared to *S. haemolyticus* JCSC1435 (85.3% vs 18.9%). Thus, ancient *Staphylococcus* strains MMP1 and MMP2 were assigned to *S. warneri* and *S. hominis* species, respectively [34]. A phylogenetic tree was built including several known *S. warneri*, *S. pasteuri*, *S. hominis*, and *S. haemolyticus* strains, and the two examined ancient strains, on the basis of 16S rRNA sequences (Figure 4). The ancient strains revealed close relations to the modern ones within species.

### Annotation

Two contigs (#23: 4058 bases; and #24: 4946 bases) from *S. warneri* MMP1 genome and three contigs (#51: 2458 bases; #53: 2908 bases; and #54: 2458 bases) from *S. hominis* MMP2 genome were recognized as plasmids by PlasmidFinder. An additional alignment test showed that all five plasmids were complete and circular.

The genome annotation results obtained by NCBI PGAAP indicate a close relation of features of ancient *Staphylococcus* strains to those of modern reference strains (Table 3).

**Table 3.** Annotation of *Staphylococcus* (*S*.) strains.

Feature	*S. warn*. MMP1	*S. warn*. SG1	*S. past*. SP1	*S. hom*. MMP2	*S. hom*. C80	*S. haem*. JCSC1435
Genome length (Mb)	2.44	2.49	2.56	2.21	2.27	2.69
G+C content	32.5	32.7	32.7	31.3	31.3	32.8
Gene count	2375	2381	2470	2193	2255	2580
Protein count	2252	2285	2377	2070	2128	2457
rRNA	4	16	16	6	21	16
tRNA	59	58	46	57	58	60
Pseudogene	60	22	31	60	48	47

*S. warn., Staphylococcus warneri; S. past., S. pasteuri; S. hom., S. hominis; S. haem., S. haemolyticus*; MMP1, Mammoth Mountain Permafrost 1; MMP2, Mammoth Mountain Permafrost 2; G+C, guanine and cytosine; rRNA, ribosomal RNA; tRNA, transfer RNA.

Whole Genome Shotgun projects have been deposited at DDBJ/EMBL/GenBank: LNVI00000000 for MMP1 and LNTW00000000 for MMP2. The versions described in this article are LNVI01000000 (MMP1) and LNTW01000000 (MMP2).

### Antibiotic resistance and virulence genes

Staphylococci are known to be resistant to different groups of antibiotics [35]. The ancient strains were tested for genes, the presence of which is known to be associated with antibiotic resistance, using ResFinder. *Staphylococcus warneri* MMP1 showed no resistance genes and *S. hominis* MMP2 possessed multiple genes associated with resistance to aminoglycoside, beta-lactam, MLS (macrolide, lincosamide, and streptogramin B), and phenicol antibiotic groups (Table 4). All identified genes showed at least 99% identity and 100% length query (except for the *blaZ* gene and *S. hominis* MMP2, with 96% identity and 100% length query).

**Table 4.** Patterns of resistance genes in *Staphylococcus* (*S*.) strains.

Antibiotic group	Resistance genes	*S. warn*. MMP1	*S. warn*. SG1	*S. past*. SP1	*S. hom*. MMP2	*S. hom*. C80	*S. haem*. JCSC1435
Aminoglycoside	*aac(6*’*)/aph(2*”)						+
	*aph(3*’*)-III*		+		+		
	*ant(6)-Ia*		+		+		
Beta-lactam	*blaZ*		+		+		+
	*mecA*						+
Fosfomycin	*fosB*						+
Fusidic acid	*fusB*			+		+	
MLS	*msr(A)*				+	+	+
Macrolide	*erm(C)*						+
	*mph(C)*				+		+
Phenicol	*cat(pC194)*				+		

Genomic DNA of Mammoth Mountain Permafrost 1 (MMP1) and Mammoth Mountain Permafrost 2 (MMP2) strains was isolated on 15 February 2010.

*S. warn., Staphylococcus warneri; S. past., S. pasteuri; S. hom., S. hominis; S. haem., S. haemolyticus*; MMP1, Mammoth Mountain Permafrost 1; MLS, macrolide, lincosamide, and streptogramin B.

Some *Staphylococcus* species are known to be pathogenic [36]. We examined the *Staphylococcus* strains using VirulenceFinder and no virulence genes were identified in either the representative or the ancient genomes.

## Discussion

It used to be thought that microorganisms present in the permafrost were mostly in a dormant or dead state. However, there is a growing body of evidence that various microorganisms isolated from permafrost and glacier ice are capable of growing, i.e. they show metabolic activity. Hence, permafrost may contain a vast number of viable microorganisms that may have a tremendous impact on a biosphere. Studies on permafrost microbial communities may lead to potentially important biotechnological applications, such as cold-tolerant enzymes [37].

A significant number of viable microorganisms from permafrost has been isolated from several Arctic and Antarctic sites. These include aerobic heterotrophs, and nitrogen-fixing, sulfur-oxidizing, sulfur-reducing, and anaerobic bacteria. These microorganisms are psychrotrophic, mesophilic, and thermophilic. For example, aerobic heterotrophic plate counts were detected in 192 out of 220 samples (87%) from nine Siberian permafrost collections [38].

The 16S rRNA from a number of permafrost microorganisms has been amplified and sequenced. Members of three major lineages were found: Gammaproteobacteria (mostly Xanthomonadaceae), Actinobacteria, and Firmicutes. The bacteria also included Actinomycetales (Arthrobacter and Microbacteriaceae), followed by Firmicutes (*Exiguobacterium* and *Planomicrobium*), Bacteroidetes (*Flavobacterium*), Gammaproteobacteria (*Psychrobacter*), and Alphaproteobacteria (*Sphingomonas*). Both culture and culture-independent approaches showed the presence of high and low guanine and cytosine (G+C)-content Gram-positive bacteria and *Gammaproteobacteria* [15,39].

Some of the 16S rRNA gene sequences of environmental clones matched those of *Arthrobacter* isolates. Two-thirds of the isolates grew at −2.5°C, indicating that they are psychroactive, and all are closely related to phylogenetic groups with strains from other cold environments, most commonly from Antarctica. The culturable and non-culturable microorganisms found in the terrestrial permafrost provide a prototype for possible life on the cryogenic planets of the Solar System [17,40].

The ability to recover viable bacteria from permafrost depends strongly on the age of the permafrost. The number and variety of microorganisms diminish with the increasing age of the permafrost. There is probably an upper time limit to the viability of microorganisms [21].

In the present paper, we report on the isolation and culturing of two ancient *Staphylococcus* strains, named MMP1 and MMP2. Genomes of the ancient bacteria were sequenced (with coverage of 40–60×) and annotated. Analysis of the sequences allowed us to classify the examined bacteria as *S. warneri* MMP1 and *S. hominis* MMP2.

Both 16S rRNA and whole genome analyses of the ancient *Staphylococcus* strains indicated their close genetic affinity with today’s *Staphylococcus* isolates. *Staphylococcus* MMP1 and *S*. MMP2 16S rRNAs were assigned to branches with modern ones within the species and 86–94% of reads obtained for the ancient strains were successfully mapped to appropriate representative genomes. A previous study showed that 2.3- and 1.8-billion-year-old deep-sea mud-inhabiting sulfur-cycling microbial communities were essentially identical and, furthermore, they were identical to the modern microbial biotas discovered off the coast of South America [41].

Many genes and gene mutations are known to be associated with bacterial pathogenicity or antibiotic resistance [42,43]. We performed a search for such genes in the examined ancient strains to elucidate whether these genes were present in *Staphylococcus* genomes 3 million years ago.

*Staphylococcus* species are divergent in their pathogenicity [36], and within species, patterns of virulence genes can be drastically different [44]. It is known that *S. warneri* and *S. hominis* species are not pathogenic [36] and we showed that this was also likely to be true 3 million years ago, as no virulence genes were identified in either of these ancient *Staphylococcus* strains.

As well as patterns of virulence genes, patterns of antibiotic resistance genes vary from strain to strain [45]. For *S. warneri* MMP1, no resistance genes were observed. However, for *S. hominis* MMP2, multiple genes associated with resistance to different groups of antibiotics were identified. It has been shown that 30,000-year-old bacteria also contained antibiotic resistance genes in their genomes [46], and evidence has been presented in support of this finding [47]. Our results strengthen the hypothesis that antibiotic resistance is an ancient feature.
